# Postoperative Recovery Time in Inguinal Herniotomy Under Ilioinguinal/Iliohypogastric Block and Sedation Versus General Anesthesia: A Retrospective Propensity-Score Matched Study

**DOI:** 10.7759/cureus.28745

**Published:** 2022-09-03

**Authors:** Cheng Lin, Edward Noh, Philip Stamov, SeonHo Jang, Kamal Kumar

**Affiliations:** 1 Anesthesiology, Western University, London, CAN; 2 Schulich School of Medicine and Dentistry, Western University, London, CAN

**Keywords:** fast track, anesthesia recovery period, ambulatory surgery, inguinal hernia repair, ultrasound guided regional anesthesia

## Abstract

Background

Associated advantages of ilioinguinal/iliohypogastric block and sedation versus general anesthesia (GA) for inguinal hernia repair have not been reported. The use of regional anesthesia (RA) is advantageous during the COVID-19 pandemic as it eliminates the need for airway manipulation.This study aimed to determine the association between postoperative recovery time when ilioinguinal/iliohypogastric block and sedation were utilized for inguinal hernia versus GA.

Method

This single-center retrospective study used multivariable logistic regression to model the anesthetic modality as a function of age, sex, body mass index (BMI), American Society of Anesthesiologists (ASA) physical status, major comorbidities to generate a propensity score for each patient for matching.

Results

After screening 295 patients, 80 patients each in the general and regional anesthesia groups were matched.RA was associated with a 35.6 minutes (95% CI: -46.6 to -25.0) shorter total postoperative recovery time when compared to GA without the increased preoperative time and adverse outcomes.

Conclusions

Inguinal hernia repair, when performed under ilioinguinal/iliohypogastric block and sedation, was associated with reduced postoperative recovery time. This can be advantageous during the time of the COVID-19 pandemic to reduce the risk of aerosol generation and shorten hospital stay. Future research can focus on establishing a causal relationship.

## Introduction

The COVID-19 pandemic has disproportionately increased the waitlist for non-urgent surgery such as herniorrhaphy [1]. The Ontario Medical Association estimated that the backlog would take hospitals running at 120% capacity and more than two years to clear [2]. In April 2022, the wait time from referral to hernia surgery in Ontario was 186 days [3]. An ongoing shortage of human resources in healthcare has posed challenges for many hospitals to ramp up operations to clear the surgical backlog [4]. Furthermore, a longer wait time for hernia surgery may increase the risk of incarceration and emergency operation [5]. While perioperative cost varies depending on settings such as for-profit, teaching, and independent healthcare facility status, a study in 2018 determined the cost per minute in the ambulatory setting is US$ 36.14 [6]. Therefore, utilizing an anesthetic technique that facilitates faster recovery and shorter stay may allow more throughput and reduce economic burden.

The anesthetic options for inguinal hernia repair include general, spinal, or local anesthesia. General anesthesia (GA) is a popular option for tertiary and academic centers, but the need for airway manipulation may render it less desirable during the COVID-19 pandemic. While spinal anesthesia obviates airway instrumentation, it has been shown to increase the risk of urinary retention and prolonged postoperative stay [7]. Local infiltration, although lacking the above shortcomings, may demand more surgical skills and is more popular in specialized hernia centers [7,8].

Ilioinguinal and iliohypogastric nerve blocks (II/IHB) provide cutaneous coverage for surgical incisions in inguinal hernia repair [9]. While the spermatic cord or the round ligament of the uterus are not blocked by II/IHB, the discomfort resulting from retracting these structures during inguinal hernia repair can be adequately covered by moderate sedation or additional infiltration by the surgeon [8]. With this knowledge, we began to provide preoperative II/IHB and intraoperative sedation for inguinal hernia repair to avoid aerosol-generating procedures during the peaks of the COVID-19 pandemic. Between June 2020 and April 2022, we successfully provided this anesthetic technique to more than 100 patients. We were not aware of II/IHB and sedation being used as the primary anesthetic technique reported beyond the case series, and we were interested in knowing whether this technique was associated with shorter recovery time when compared to general anesthesia.

The primary objective of the study was to determine whether the combination of ilioinguinal/iliohypogastric block and sedation, when compared to GA in an inguinal hernia, is associated with reduced postoperative recovery time through a retrospective propensity score-matched cohort study.

## Materials and methods

This single-center retrospective study (ID: 119564) was approved by the Western University Health Science Research Ethics Board. Data was extracted from the institution's electronic medical record by the co-investigators - Noh, Stamov and Jang.

Population

The inclusion criteria were 18 years or older, American Society of Anesthesiologists (ASA) physical status I to III, and body mass index (BMI) less than 45. Patients were scheduled for an elective ambulatory unilateral inguinal hernia repair. The hernia defect should be less than 3 cm. The surgery was open, tension-free, and mesh repair (Lichtenstein's technique). Exclusion criteria included opioid dependence defined as daily oral morphine equivalents use of more than 30 mg, personal or family history of malignant hyperthermia, and pregnancy. Patients with incomplete postoperative recovery time data were excluded. All elective hernia repairs were screened between June 1, 2020, and April 1, 2022. All procedures took place in our tertiary academic center equipped with a block room. Laparoscopic and endoscopic hernia repairs were not offered in our institution.

Anesthesia

Block and sedation were offered as the default option, although GA was always mentioned as an alternative. For patients who received II/IHB (regional anesthesia, RA group), ultrasound-guided nerve blocks were placed in the block room, 30 minutes preoperatively, utilizing commonly described techniques [10]. A 20 mL mixture of 2% lidocaine with 1:200,000 epinephrine and 0.5% ropivacaine in a 1:1 ratio was used. The option of II/IHB and sedations were offered to all patients undergoing inguinal hernia repair when one of five regional anesthesiologists were available in the block room. Intraoperative sedation consisted of propofol infusion, targeting a Ramsay Sedation Scale (RSS) from four to five.

Patients who declined blocks and sedation or scheduled when the group of five anesthesiologists was not covering the block room would receive general anesthesia (GA group). Neuraxial anesthesia was not offered. Intravenous induction with fentanyl and propofol, without muscle relaxants, and maintenance with sevoflurane without nitrous oxide was administered. The airway was maintained with supraglottic airway devices. Surgeons would infiltrate the surgical site with 20 to 30 mL of 0.5% bupivacaine at the end of the operation.

Both groups had intravenous dexamethasone, ketorolac, and ondansetron in the operating room. Most patients received intraoperative intravenous morphine equivalents of 2 mg to 5 mg at the discretion of the operating room anesthesiologists.

Postoperatively, any patients who scored at least nine out of 10 on the modified Aldrete's score would be transferred to phase two recovery. Once there, patients who scored at least nine out of 10 on the Post Anesthetic Discharge Scoring System were discharged from the hospital. Our institution did not require patients undergoing inguinal hernia to void prior to discharge.

Measured outcomes

The primary outcome was the total postoperative recovery time, defined as when the patient left the operating room (OR) to the time the patient was ready for hospital discharge. The secondary outcomes included total preoperative time, defined as when patients registered to OR entry, operating time, i.e., the time between OR entry to OR exit, and total hospital length of stay. We recorded postoperative nausea and vomiting if patients experienced any associated symptoms or received postoperative antiemetics, excluding prophylactic doses. Severe postoperative pain was noted if patients were recorded to have at least seven out of 10 pain in the postoperative period. A postoperative desaturation episode was defined as any incidence when oxygen saturation was below 90% with or without oxygen.

Statistical methods

G*Power (version 3.1.9.7; Heinrich Heine University Düsseldorf, Germany) was utilized for power calculation. The sample size was calculated with α=0.05 and β=0.8, using a mean total recovery time of 135 minutes and a standard deviation of 61 minutes based on our institution data. We considered a 30-minute reduction to be clinically significant. Based on these parameters, 110 patients, 55 in each arm, were required. 

Statistical analysis was done using R (version 4.1.2; R Foundation for Statistical Computing, Vienna, Austria) and determined a priori. Patient demographics, including age, sex, BMI, ASA, and comorbidities, were presented in mean and standard deviation (SD) or numbers and percentages. Recorded comorbidities include cardiac diseases (coronary disease, congestive heart failure, moderate to severe valvulopathy), chronic obstructive pulmonary diseases (COPD), diabetes mellitus, and obstructive sleep apnea (OSA). Intention-to-treat analysis was performed. Propensity score matching was used to deal with potential confounders. We used multivariable logistic regression to model the anesthesia modality (GA vs. RA) as a function of age, sex, BMI, ASA, cardiac diseases, COPD, diabetes, and OSA to generate a propensity score for each patient. Age and BMI were treated as continuous variables, and sex (biological male vs. female), ASA (I and II vs. III), and each of the four comorbidities (present vs. absent) were treated as nominal categorical variables. Matching was done using the nearest neighbor method, 1:1 matching without replacement, and a caliper of 0.1. Unmatched patients were not included in the post-match analysis. The standardized difference in means (SDM) was calculated for each covariate before and after matching to assess for balance. We considered a difference of <0.10 in SDM a balanced match. Shapiro-Wilk's test was performed on the continuous outcomes to determine normality. Normally distributed continuous variables were analyzed with Student's t-test, and non-parametric continuous variables were analyzed with the Mann-Whitney U test. Fischer's exact tests were done for categorical outcomes. E-values were calculated to estimate the effect of unmeasured confounders on statistically significant post-matched outcomes.

## Results

**Figure 1:**
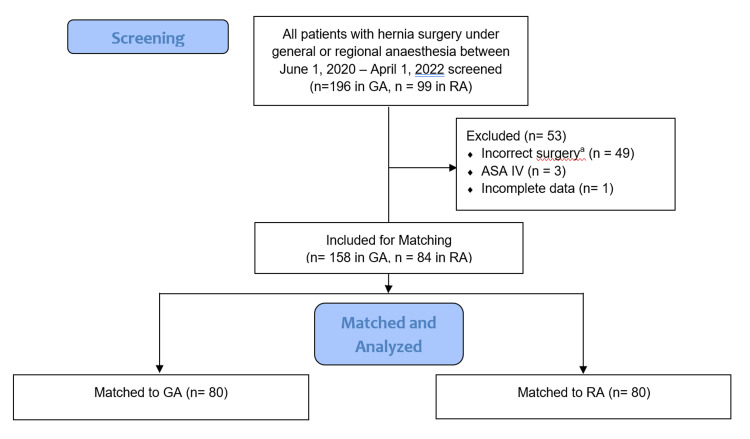
Flow diagram of cases screened, included and analyzed GA - general anesthesia; RA - regional anesthesia; ASA - American Society of Anesthesiologists ^a ^Incorrect surgery included non-inguinal hernia repair (i.e. umbilical, incisional), more than one hernia repair (i.e. bilateral or umbilical and inguinal), or combined surgery (i.e. hydrocelectomy and inguinal hernia repair)

Between June 1, 2020, to April 1, 2022, 295 patients were screened, with 242 ultimately meeting the criteria (158 in the GA group and 84 in the RA group). Eighty patients in each group were successfully matched (Figure 1). In the pre- and post-matched RA group, seven patients required conversion to general anesthesia. The reasons included block failure (3), surgeons' request (2), and coughing (2). 

Patient demographics of each group before and after the match were noted in Table 1. There was a higher proportion of male patients as expected from this type of surgery. Incidence of major comorbidities were low, reflecting the nature of day surgery population. The SDM before the match showed some heterogeneity in age, BMI, ASA, COPD, diabetes, and OSA, which were improved after the match, with all SDMs below 0.10 after matching. 

**Table 1:** Pre- and post-match baseline demographic characteristics SD - standard deviation; BMI - body mass index; ASA - American Society of Anesthesiologists; COPD - chronic obstructive pulmonary disease ^a^ Standardized differences were presented as proportion. ^b^ Biological sex ^c^ Cardiac disease included coronary disease, congestive heart failure, moderate to severe valvular diseases ^d^ Included patients on oral hypoglycemics and insulin ^e^ Included patients using and not using continuous positive airway pressure (CPAP) mask

		Pre-match			Post-match		
	General anesthesia		Regional anesthesia	Standardized difference^a^	General anesthesia	Regional anesthesia	Standardized difference^a^
Sample size, n		158	84		80	80	
Mean age in years ±SD		57.4 ± 15.0	53.6 ± 15.8	0.26	55.7 ± 14.3	54.8 ± 15.2	0.09
Sex, n (%)^b^				0.08			0.03
Male		134 (84.8)	71 (84.5)		67 (83.8)	68(85.0)	
Female		24 (15.2)	13 (15.5)		13 (16.2)	12 (15.0)	
Mean BMI ± SD in kg/m^2 ^		26.5 ± 4.5	26.0 ± 4.4	0.11	26.3 ±4.1	26.2 ± 4.4	0.03
ASA, n (%)				0.13			0.03
I and II		114 (74.1)	67 (79.8)		63 (79.8)	64 (80.0)	
III		41 (25.9)	17 (20.2)		17 (21.2)	16 (20.0)	
Comorbidities, n (%)							
Cardiac disease^c^		9 (5.7)	4 (4.8)	0.04	4 (5.0)	3 (3.8)	0.05
COPD		2 (1.3)	0 (0)	0.11	0 (0)	0 (0)	0
Diabetes^d^		7 (4.4)	1 (1.2)	0.16	0 (0)	0 (0)	0
Sleep apnea^e^		4 (2.5)	0 (0)	0.16	0 (0)	0 (0)	0

All continuous outcomes were non-parametric based on the Shapiro-Wilk test. There was a reduced total recovery time of 35.6 min (95% CI: -46.6 to -25.0) associated with RA and sedation versus GA. Secondary outcomes, including operating room time and total length of stay, also showed significant reductions of 14 minutes (-20.0 to -8.0) and 67 minutes (95% CI: -86.0 to -46.8), respectively, where RA and sedation were the associated technique. Other outcomes, including preoperative time, the incidence of postoperative nausea and vomiting, and severe pain, were not different between the two techniques (Table 2). No patient experienced a desaturation episode in the postoperative period in either group. E-values, including a 95% confidence interval, are presented in Table 2.

**Table 2:** Measured outcomes in pre- and post-matched samples with difference in mean and odds ratios IQR - interquartile range; ASA - American Society of Anesthesiologists; COPD - chronic obstructive pulmonary diseases; OSA - obstructive sleep apnea ^a ^Outcomes under pre-matched samples were unadjusted, presented as median and IQR, or number and percentage ^b ^Outcomes from matched samples were adjusted from a multivariable regression model taking age, BMI, sex, ASA, cardiac disease, COPD, diabetes, OSA as co-variates, presented as median and IQR, or number and percentage ^c ^p-value compared post-matched samples between general anesthesia and regional anesthesia groups, using p<0.05 as a statistically significant ^d^ Mann-Whitney U test was used for continuous outcomes and Fisher's exact test for categorical outcomes

	Pre-match^a^			Post-match^b^				
	General anesthesia		Regional anesthesia	General anesthesia	Regional anesthesia	Mean difference (95% CI)	p-value^c, d ^	E-value (95% CI)
Total recovery time in minutes [IQR]	118.0 [99.3 – 147.3]		84.0 [64.0 – 112.0]	119.0 [99.8 – 150.0]	84.0 [66.0 – 112.2]	-35.6 (-46.6 to -25.0)	<0.001	3.59 (2.48 – 4.69)
Preoperative time in minutes [IQR]	111.5 [77.0 – 144.8]		100.0 [72.3 – 122.5]	112.5 [76.5 – 136.8]	101.5 [74.8 – 122.5]	-10.0 (-24.0 to 4.0)	0.12	
Operating time in minutes [IQR]	70.0 [53.3 – 86.0]		54.0 [47.0 – 64.3]	72.0 [53.8 – 86.0]	54.0 [46.8 – 64.0]	-14.0 (-20.0 to -8.0)	<0.001	3.35 (2.31 – 4.40)
Total length of stay in minutes [IQR]	308.5 [274.0 – 355.8]		249.0 [206.2 – 355.8]	310.0 [274.8 – 342.5]	251.5 [206.2 – 297.7]	-67.0 (-86.0 to -46.8)	<0.001	4.32 (3.02 – 5.61)
Adverse events, n (%)						Odds ratio (95% CI)		
Nausea or vomiting	12 (7.6)	3 (3.6)		7 (8.8)	3 (3.8)	0.31 (0.05 – 1.30)	0.13	
Severe pain	12 (7.6)	7 (8.3)		8 (10.0)	7 (8.8)	1.43 (0.44 – 6.00)	0.77	
Desaturation	0 (0)	0 (0)		0 (0)	0 (0)			

## Discussion

Utilizing regional anesthesia and sedation in place of general anesthesia has been shown to have many benefits in the literature. This included reduced time spent in the operating room, recovery and total hospital length of stay [11,12]. While not demonstrated in our study, other benefits of regional anesthesia include reduced perioperative opioid consumption, avoidance of general anesthesia, and, consequently, lower incidence of postoperative nausea and vomiting [12,13]. Local or non-neuraxial regional anesthesia, when used as the primary technique in inguinal hernia repair, may reduce the risk of urinary retention [14,15]. Furthermore, regional anesthesia obviates the need to instrument the airway. The current meta-analyses on GA versus local anesthesia showed a higher risk of urinary retention, high pain score, longer time to return to activity, and similar patient satisfaction in the GA group, and no specific recovery advantage of GA was identified if the hernia was amenable to any anesthetic technique [16,17]. In our experience, GA may be a more favorable option for morbidly obese patients, patients with sleep apnea, or surgical requirements such as paralysis or massive hernia size.

There could be multiple reasons for the shorter perioperative stays. Regional anesthesia and sedation eliminated the need for induction, airway instrumentation, and emergence, permitting surgeons to proceed as soon as the patient arrived in the operating room and, likewise, allowing patients to leave the operating room as soon as the surgery was finished. As patients were lightly sedated, many would have scored at least nine out of 10 on the modified Aldrete's score, allowing a faster journey through recovery.

Truncal blocks, such as ilioinguinal/iliohypogastric blocks or transversus abdominis plane (TAP) blocks, have traditionally been utilized as an analgesic component in addition to general anesthesia due to their incomplete surgical coverage for inguinal hernia repair [18 ]. With moderate sedation, the visceral discomfort caused by retraction was adequately tolerated in our study, with seven out of 84 patients requiring conversion to general anesthesia. The depth of sedation may be further reduced with local anesthetic infiltration at the spermatic cords or round ligament of the uterus [8]. Chronic groin pain following inguinal hernia repair may be as high as 63%, with a proportion being neuropathic pain in the ilioinguinal, iliohypogastric, or genitofemoral nerve distribution. Practitioners should exercise caution as an ilioinguinal/iliohypogastric nerve block in this setting may expose one to the risk of litigation [19]. The incidence of transient femoral nerve palsy following ilioinguinal block is reported at a rate of 2.6-6%, and quadriceps weakness may delay hospital discharge [20,21]. Finally, sensory distributions of ilioinguinal and iliohypogastric nerve show inter-individual variation, which may explain why some cases required conversion to GA in addition to the block failure [9].

Hadzic et al. utilized a multilevel paravertebral block and sedation for inguinal hernia repair and showed shorter hospital length of stay, faster return to ambulation, and reduced pain when compared to GA [22]. The technique described requires patient re-position and multiple injections. There is also the potential of the rare but serious risk of pneumothorax. Compared to paravertebral blocks, ilioinguinal/iliohypogastric blocks are more technically accessible to non-subspecialized anesthesiologists.

Factors shown to delay hospital discharge after day surgery include female sex, elderly (>80 years old), congestive heart failure, spinal anesthesia, postoperative nausea vomiting (PONV), pain, ASA III or IV, longer duration of surgery, and poor social support [12,23]. While we had included many of these factors in our regression model, we did not adjust for an intraoperative time as we would like to determine if RA was associated with a shorter time in the OR. Awad et al. suggested that the delayed discharge related to longer surgical duration may be due to a higher risk of PONV and pain [12]. Our matched sample showed a similar incidence of PONV and severe pain between both groups, and the majority of cases were performed within 90 minutes based on the interquartile ranges of intraoperative time. Factors that were unavailable on our electronic record that may still influence discharge time include social support, preoperative education, and hydration status [12,24]. Taken together, with the E-value of 3.59 (95% CI: 2.48-4.69) for total recovery time, we believe the primary outcome was robust against unmeasured confounders. Another limitation of our study was a reduced sample size after propensity score matching. However, increased homogeneity between the matched groups may increase the strength of association between anesthesia type of postoperative recovery time. II/IHB may be considered a moderately challenging block, requiring trained expertise for placement [25]. The presence of a block room was also important for this surgical pathway. As a single-center study, our surgeons may also adopt surgical techniques more suitable for regional anesthesia and sedation.

## Conclusions

Using sedation and ilioinguinal/iliohypogastric block for open inguinal hernia repair can be an attractive alternative to general anesthesia. This moderately challenging block and the requirement for a block room may limit the wide adoption of our technique. Our propensity score-matched cohort study showed that this technique was associated with a reduced postoperative recovery time, intraoperative time, and total length of hospital stay without an increase in preoperative time. No difference was detected in the incidence of nausea and vomiting nor severe pain between regional anesthesia with sedation and general anesthesia.
